# Asthma diagnosis and treatment – 1023. The implementation of asthma management guideline and the obstacle factors in Korea

**DOI:** 10.1186/1939-4551-6-S1-P22

**Published:** 2013-04-23

**Authors:** Eun-Jung Jo, Mi Yeoung Kim, Suh Young Lee, Seoung-Eun Lee, Woo-Jung Song, Sae-Hoon Kim, Sang-Heon Cho, Kyung-up Min, You-Young Kim, Yoon-Seok Chang

**Affiliations:** 1 Internal Medicine, Seoul National University Bundang Hospital, Seongnam, South Korea; 2 Internal Medicine, Seoul National University Hospital, Seoul, South Korea; 3 Allergy and Asthma, National Medical Center, Seoul, South Korea; 4 Department of Internal Medicine, Seoul National University College of Medicine, Seoul, South Korea

## Background

There is gap between the guideline and real practice of asthma management. The implementation of asthma management guideline is essential to reduce the gap and for the qualified standard care. We evaluated the implementation of asthma management guideline and obstacle factors to the implementation in Korean physicians.

## Methods

From March to April 2012, a total of 165 physicians in primary care, secondary and tertiary hospitals were enrolled. They filled in a questionnaire about their current practice on asthma: whether they followed the management guideline and if not, what might be the obstacle factors.

## Results

Thirty eight percent of the physicians were male and their mean age was 43 (± 8) years old. Ninety five percent of physicians had asthma patients in their clinics. Most of them (83.2%) knew about the asthma management guideline and 87.4% of them used the guideline on their asthma practice. Among the physicians, one hundred and twenty two (73.9%) were primary care physicians. 65.6% of the primary care physicians answered that they practiced according to the guideline for more than half of their patients. They reported difficulties in monitoring asthma control status. Only 26.2% of the primary care physicians prescribed inhaled corticosteroid (ICS) to most of their asthma patients, and the reasons that they do not prescribe ICS that much were physicians’ preference for oral medications and the concern about the possible refusal by the health insurance.

## Conclusions

In primary care physicians, there was a huge gap between the management guideline and real practice. This study shows the necessity of education that ICS is the first-line choice in the treatment of asthma, and strongly suggests that the current health insurance policy should be improved for the better asthma care.

